# Origins of Ripples in CVD-Grown Few-layered MoS_2_ Structures under Applied Strain at Atomic Scales

**DOI:** 10.1038/srep40862

**Published:** 2017-01-19

**Authors:** Jin Wang, Raju R. Namburu, Madan Dubey, Avinash M. Dongare

**Affiliations:** 1 Department of Materials Science and Engineering and Institute of Materials Science, University of Connecticut, Storrs, Connecticut 06269, USA; 2 Computational and Information Sciences Directorate, U.S. Army Research Laboratory, Aberdeen Proving Ground, Maryland 21005, USA; 3 Sensors and Electron Devices Directorate, U.S. Army Research Laboratory, Adelphi, Maryland 20783, USA

## Abstract

The potential of the applicability of two-dimensional molybdenum disulfide (MoS_2_) structures, in various electronics, optoelectronics, and flexible devices requires a fundamental understanding of the effects of strain on the electronic, magnetic and optical properties. Particularly important is the recent capability to grow large flakes of few-layered structures using chemical vapor deposition (CVD) wherein the top layers are relatively smaller in size than the bottom layers, resulting in the presence of edges/steps across adjacent layers. This paper investigates the strain response of such suspended few-layered structures at the atomic scales using classic molecular dynamics (MD) simulations. MD simulations suggest that the suspended CVD-grown structures are able to relax the applied in-plane strain through the nucleation of ripples under both tensile and compressive loading conditions. The presence of terraced edges in these structures is the cause for the nucleation of ripples at the edges that grow towards the center of the structure under applied in-plane strains. The peak amplitudes of ripples observed are in excellent agreement with the experimental observations. The study provides critical insights into the mechanisms of strain relaxation of suspended few-layered MoS_2_ structures that determine the interplay between the mechanical response and the electronic properties of CVD-grown structures.

The 2-dimensional (2D) structures of MoS_2_ exhibit significantly different electronic band structure from bulk MoS_2_ structures^1^,^2^ and therefore show significant promise for applications in field effect transistors^3^, optoelectronics^4^, photo transistors and detectors^5^, and chemical catalysts^6^,^7^. The variations in the electronic structure of 2D MoS_2_ structures under the application of external strain^8^,^9^,^10^,^11^ open up the possibility to tune the performance of the material for device applications. The optimization of the applicability of 2D MoS_2_ as a building block in future electronic devices hinges on a fundamental understanding of the stability and variations in the electronic properties of 2D MoS_2_ structures as well as the microstructural response of 2D MoS_2_ structures as a function of strain. As a result, a significant number of experimental^12^,^13^,^14^,^15^,^16^,^17^,^18^ and theoretical^13^,^19^,^20^,^21^,^22^ studies have investigated the strain response of mechanically exfoliated 2D MoS_2_ monolayer (ML) and few-layered (FL) structures.

The mechanical response of these 2D materials shows exceptionally high strengths under tension. For example, nanoindentation experiments using an atomic force microscope show that ultrathin free-standing MoS_2_ membranes have an effective Young’s modulus of 250 ± 150 GPa and may bear a large in-plane strain up to 11% before fracture, implying a broad operation range for strain engineering in 2D MoS_2_ materials^23^. In addition, structural ripples have been frequently observed as a strain relaxation mechanism in 2D materials, such as graphene^24^, ML and FL MoS_2_^25^, and other van der Waals (vdW) layered materials^26^. The formation of ripples results in the degradation of mobility of the carrier mass and alteration of electronic structures in these 2D materials. Buckling and ripple formation has also been predicted using molecular dynamics (MD) simulations under uniaxial compression^27^. The size of the ripples generated during compression is observed to be associated with the strain rate and length of the sample^28^.

Recently, the capability to grow high quality and large areas of ML and FL MoS_2_ sheets for device applications has been made possible through chemical vapor deposition (CVD)^29^,^30^,^31^. These as-grown 2D terrace structures comprise of flakes of varying number of layers wherein the top layers are relatively smaller in size than the bottom layers, resulting in the formation of terrace regions and edges/steps across adjacent layers. The presence of these edges is likely to affect the observed strain-induced electronic and mechanical response of 2D MoS_2_ structures that do not include such edges. The strain response of such mechanically exfoliated FL-MoS_2_ structures with no edges is characterized by shifts in both the Raman in-plane mode and photoluminescence (PL) energy.

The strain response of the CVD-grown terraced MoS_2_ structures is determined by the presence of edges and also results in slippage between the layers^32^. Recent DFT simulations suggest that the response of such 2D structures (with unequal dimensions) is likely to create an inequality in the amounts of strain across the layers and hence exhibits inhomogeneous electronic properties^33^. In addition, ripples are observed near edge terminals in CVD-grown MoS_2_ structures using scanning tunneling microscopy (STM)^34^. These ripples, with a typical height of ~11 Å, are reported to be responsible for the reduction of band gap of the MoS_2_ sheets near edge terminals from 1.96 eV to 1.46 eV. A recent study^35^ suggests the formation of ripples in as-grown bilayer MoS_2_ structures that is induced by strong interlayer edge-to-basal plane coupling. In addition, the rippling instability is observed in the lower layer due to the interactions with the edges in the top layer.

Thus, while the strain response of MoS_2_ structures has been investigated extensively for the case of mechanically exfoliated flakes, the understanding of the effects of strain on the mechanical and electronic behavior of CVD-grown MoS_2_ structures is still in infancy. Although DFT simulations can provide insights in the effect of the presence of edges on the strain-induced variations in the local electronic structure of such multilayered structures, these simulations are limited to a system size on the order of a few nanometers. It is also likely that the effect of the presence of edges on the strain response observed at the nanoscale dimensions of the layers is significantly different at the micron scale dimensions of the layers as observed experimentally. Micron scale exceeds the capacity of DFT method, yet can be handled using classical MD. Moreover, the DFT simulations focused on the strain response of bilayer structures, hence, the effect of number of layers as well as the mechanism of strain relaxation at the atomic scale remain unknown.

The current study investigates the effect of the size of the layers and mechanism of the strain relaxation of suspended CVD-grown multilayered MoS_2_ structures at the experimental length scales using classical MD simulations. The CVD-grown structures comprise of flakes of few layers wherein the top layers are relatively smaller in size than the bottom layers, resulting in the formation of edges/steps across adjacent layers. To model such a system, a simplified five-layered terraced MoS_2_ rectangular system in the basal plane with edges along X direction is created and shown in Fig. 1. All the layers have the same width in the Y direction (~11 nm), but have varying dimensions in the X direction. The CVD-grown terraced pyramid structure is created to have a 1/5 decrease in the length of each layer in the X direction as compared to the length of the layer below it. The X dimensions of the as-created pyramid system correspond to ~317 nm for the bottom-most layer (layer 1), ~254 nm for layer 2, ~203 nm for layer 3, ~164 nm for layer 4, and ~130 nm for layer 5. The thickness of each individual layer is 0.62 nm from our simulation, which leads to 3.1 nm for the five-layer system. The total dimensions of the representative system are 317 nm × 10.98 nm × 3.1 nm. Periodic boundaries are applied in X and Y directions, and a vacuum space of 20 nm is added in the vertical direction. Such a simplified system creates edges along the X direction, and allows the investigation of the strain response of a particular layer when the free edges may appear both above and below it. The interatomic interactions are defined by a combined reactive empirical bond-order (REBO)^36^ and Lennard Jones (LJ) potential. The strain response is investigated under the loading condition of uniaxial tensile strain (*ε*_*x*_ ≠ 0, *ε*_*y*_ = 0, *σ*_*x*_ ≠ 0, and *σ*_*y*_ ≠ 0,) where *ε*_*x*_ and *ε*_*y*_ (*σ*_*x*_ and *σ*_*y*_) are the *average* strains (stresses) of the entire system along the X and Y directions.

## Results and Discussions

A uniaxial tensile strain is applied along the X direction in increments of 0.1% and the system is allowed to relax to minimize the total energy of the system at each increment. The straining is continued to reach a total tensile strain of 6% which is within the elastic region^37^ for the MoS_2_ system for the REBO potential. At each increment, a layer strain (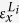) is calculated for each individual layer as


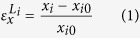


where *i* is the layer-id (1, 2, 3, 4, 5); *x*_*i*_ and *x*_*i*0_ are final and initial layer length of *i*^th^ layer in X direction, respectively. The analysis of the layer strain, 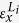, provides the strains of each individual layer and enables the investigation of the relaxation of each layer during the straining process. The variating of 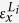 is plotted as a function of the applied uniaxial tensile strain (*ε*_*x*_) for each individual layer, from layer 1 to layer 5, in Fig. 2. It can be seen that the layer strain in the bottom layer varies as the applied external strain. No relaxation of the bottom layer is observed as there are no edges on the bottom layer. However, the variation in the layer strain for the upper layers i.e. layers 2 through 5, is observed to deviate from that observed for the bottom layer with the layer strains being lower than that for the bottom layer. The deviations are observed to be earlier and larger for the higher layers. Such a deviation suggests that the top layers start to relax first due to the presence of a free surface above the top layer, followed by the lower layers. The deviation in the layer strain, therefore, is larger for the upper layers where vertical relaxation is possible. The layer strain continues to increase with applied strain for each layer up to a certain value, after which a further increase in applied strain results in a drop in the local strain. These reductions in layer strain are observed to occur simultaneously for layer 2 through layer 5. Each reduction is then followed by a small increase in layer strain when strained further. Such increases and decreases in the applied strains continue to occur simultaneously as the applied strain increases, but the deviation in the magnitude of the layer strains is also observed to increase as the applied strain increases. For example, at an applied strain of *ε*_*x*_ = 4%, the layer strains, from layer 2, layer 3, layer 4 and layer 5 reduce to 3.29%, 3.06%, 2.62%, 1.33%, respectively. However, it is not clear if these simultaneous variations are related to the sliding between the layers or due to the slipping of the edges on top of the layers.

**Figure 1:**
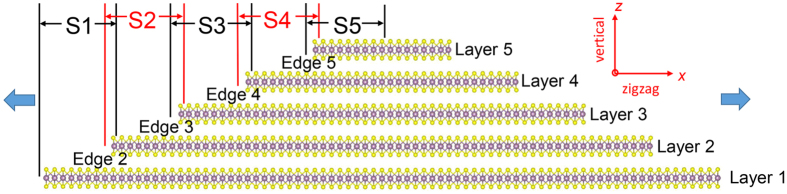
Schematic of a model pyramid system used in the study. Uniaxial strain is applied along the X direction as indicated by a blue arrow.

To investigate the strain relaxation mechanisms of the various layer strains, an out-of-plane vertical displacement is computed for each atom along the length of layers in the multilayered structure. This vertical displacement (δ*z*_*i*_), at a certain atom *i*, is computed as δ*z*_*i*_ = *z*_*i*_−*z*_0*i*_, where *z*_*i*_ and *z*_0*i*_ are the instantaneous and initial Z-coordinate of atom *i*, respectively^24^,^35^,^38^. The vertical displacement will be expected to be uniform for the case of sliding between the layers as the strain relaxation mechanism. For the case of the slipping of the edges on the bottom layers, a vertical displacement profile will show high displacements at the edges of each layer. The visual analysis of these vertical displacements for the entire sample, however, become challenging due to the large X-to-Z ratio (2D structure) of the pyramid structure. As a result, the discussion is now focused on only the left half of the structure along the X direction as the structure is symmetric on both sides. The left half of the pyramid sheet is partitioned to five sections, S1 to S5, to visualize the local variations in vertical displacements at the atomic scale, as shown in Fig. 1. The five sections overlap with each other to include the terrace and an edge. A total number of four edges are investigated in this paper, noted as E2 to E5. Figure S3 shows the displacement map for sections S1 to S5 of the left half of the MoS_2_ pyramid at unstrained configuration. The atoms are colored by the displacement in the Z direction with the positive value (red color) corresponding to an upward displacement and a negative value (blue color) indicating a downward displacement as compared to the unstrained configuration. At strain-free configuration (*ε*_*x*_ = 0%), there is no displacement along vertical direction for all atoms. Therefore, δ_*zi*_ of all the atoms equals to zero and these atoms are colored in green, as shown in Figure S3.

**Figure 2:**
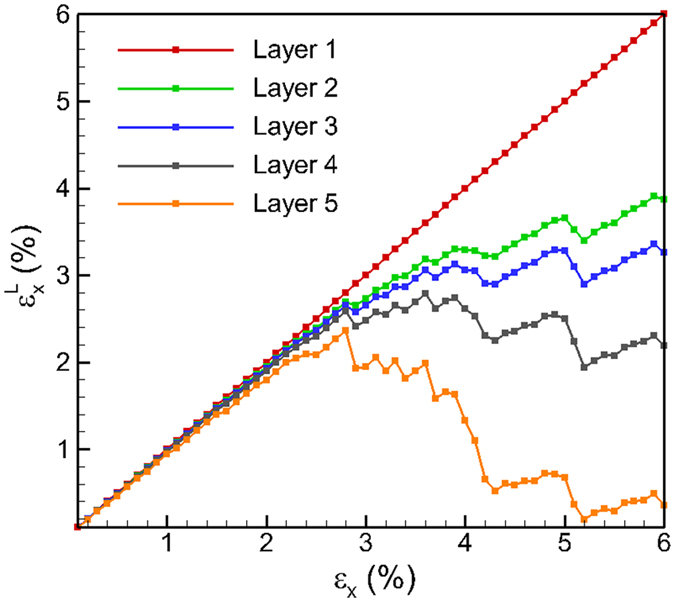
Variating of layer strain 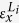 for each layer as a function of applied strain *ε*_*x*_ for 317 nm MoS_2_ multilayered structure under uniaxial tension.

**Figure 3:**
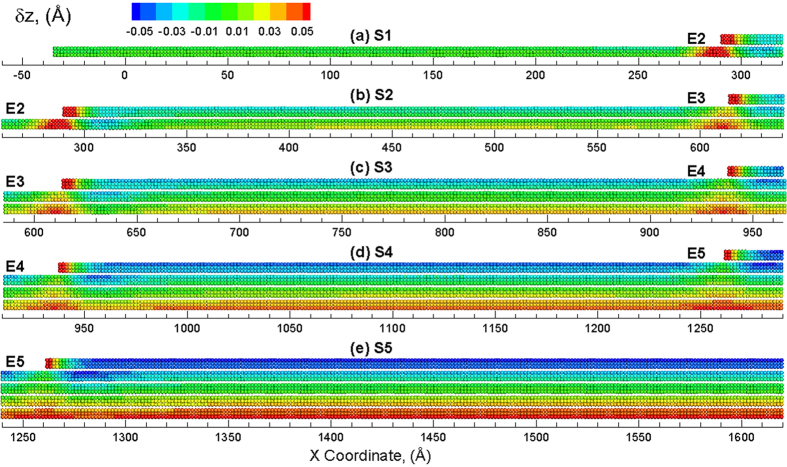
Snapshots of different sections (**a**) S1, (**b**) S2, (**c**) S3, (**d**) S4 and (**e**) S5 of the multilayered structure at *ε*_*x*_ = 2% showing formation of ripples at the edge region. The atoms are colored by the displacement in the Z direction with the positive value (red color) corresponding to an upward displacement and a negative value (blue) indicating a downward displacement as compared to the unstrained configuration.

The vertical displacements can now be applied to investigate the response of the pyramid structure. The vertical displacements in the various sections of the pyramid structure at an applied strain of *ε*_*x*_ = 0.1% are shown in Figure S4. As can be seen in the snapshots, the top layer (sulfur atoms) of each section is displaced downwards (blue) and the bottom layer (sulfur atoms) is displaced upwards. It can be seen that the displacement is uniform along the length of each section and the maximal displacement is computed to be ~ 0.0005 Å at an applied strain of *ε*_*x*_ = 0.1%. The uniform straining of the layers continues to increase up to an applied strain of *ε*_*x*_ = 2%, wherein a deviation is observed in the layer strains in Fig. 2. The vertical displacements in the various sections of the pyramid structure at an applied strain of *ε*_*x*_ = 2% are shown in Fig. 3. It can be seen from the snapshots that a vertical displacement is observed at the edges in each of the sections. This vertical displacement of the edge also results in a vertical displacement of the layers below the edge region as suggested by the red colored atoms. This vertical displacement of the edge region is also compensated by having a slight downward displacement (as shown by blue color) in the region next to the edge region. This upward/downward displacement of the edges, as will be discussed later, results in the nucleation of ripples that result in the variations in the layer strains plotted in Fig. 2. The generated ripple can be observed at the right side of E2 as shown in Fig. 3(b). The ripple is more visible at the right side of E3 (Fig. 3(c)), E4 (Fig. 3(d)) and E5 (Fig. 3(e)). No ripple is nucleated in S1, as indicated by Fig. 3(a). This strain (~2%) coincides with the strain at which the local layer strain of top layers starts to deviate the applied strain (as discussed before). As a result, the relaxation of the top layers may be attributed to the ripples induced by the edge.

**Figure 4:**
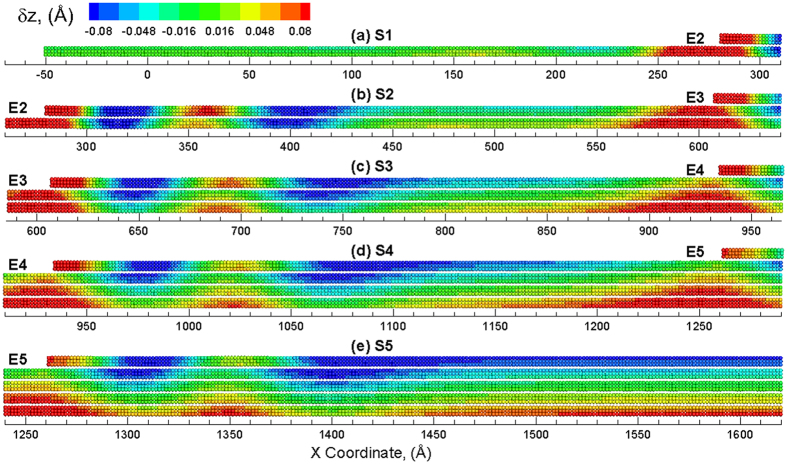
Snapshots of different sections (**a**) S1, (**b**) S2, (**c**) S3, (**d**) S4 and (**e**) S5 of the multilayered structure at *ε*_*x*_ = 3% showing propagation of ripples from edge towards center. The atoms are colored by the displacement in the Z direction with the positive value (red color) corresponding to an upward displacement and a negative value (blue) indicating a downward displacement as compared to the unstrained configuration.

**Figure 5:**
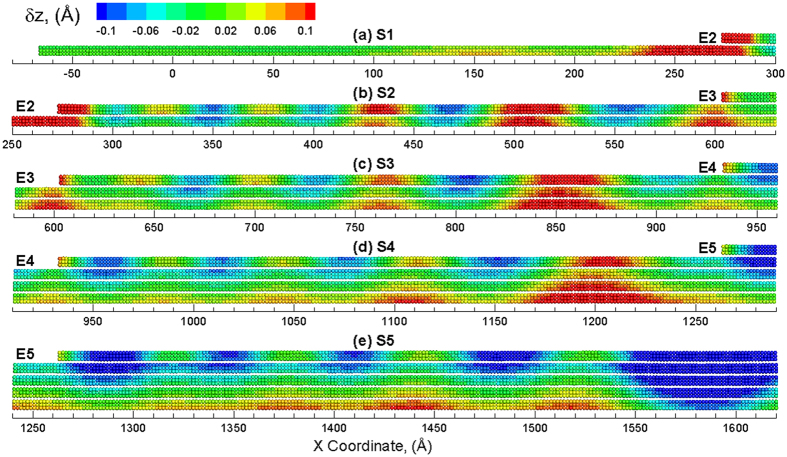
Snapshots of different sections (**a**) S1, (**b**) S2, (**c**) S3, (**d**) S4 and (**e**) S5 of the multilayered structure at *ε*_*x*_ = 4% showing ripples across the length of each section. The atoms are colored by the displacement in the Z direction with the positive value (red color) corresponding to an upward displacement and a negative value (blue) indicating a downward displacement as compared to the unstrained configuration.

Further straining of the structure results in propagation of the nucleated ripples as well as nucleation of new ripples. The vertical displacements in the various sections of the pyramid structure at an applied strain of *ε*_*x*_ = 3% are shown in Fig. 4. As shown in Fig. 4(a), ripples are still not seen in section S1 as the strain increases to 3%. However, another ripple is nucleated at the edge region in other four sections (Fig. 4(b) to (e)). All of these ripples propagate from edge region towards the center of the structure, i.e., from left towards right. The number of ripples propagating in each section, i.e. 2 at *ε*_*x*_ = 3%, is the same. It should be noted that the top layers in each section show significant downward displacement (suggested from more blue atoms) as compared to the remaining layers in each section. This displacement attributed to the edges of the top layers results in the reduction in the layer strain for the top layers, as seen in Fig. 2. An applied strain of *ε*_*x*_ = 4% results in the propagation of ripples across the length of the sections as shown in Fig. 5. The number of ripples propagating in each section is the same except for that observed in section S1, where the ripples only propagate towards the half way for an applied strain of *ε*_*x*_ = 4%. However, five ripples are observed (indirectly implied by number of blue regions in the area) to be propagating in section S2. For sections S3, S4 and S5, five propagating ripples can be explicitly observed. It is also interesting to note that the ripples generated in section 4, propagate along the length of the section and interact with the edge (E5) in section 5 where ripples are nucleated in section 5. For the section S5 which belongs to the left half of the system, ripples are also generated in the right half of the system due to the edge that propagate towards the center of the multilayered structure i.e. from right to left. The ripples created meet and interact at the center of the system to create a large downward displacement (blue) region at the center of the multilayered system as shown in Figure S5(a). Further strain results in the propagation of this downward displacement region in the opposite direction of the ripples as shown in Figure S5(b). The application of more strain on the structure results in the nucleation of new ripples at the edges that propagate again towards the edges as shown in Figure S5(c).

**Figure 6:**
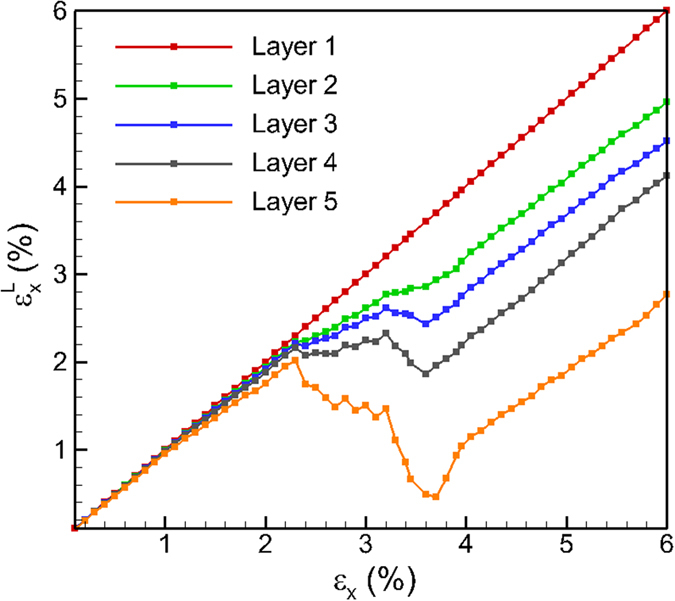
Variating of layer strain 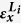 for each layer as a function of applied strain *ε*_*x*_ for 317 nm MoS_2_ multilayered structure under uniaxial compression.

**Figure 7:**
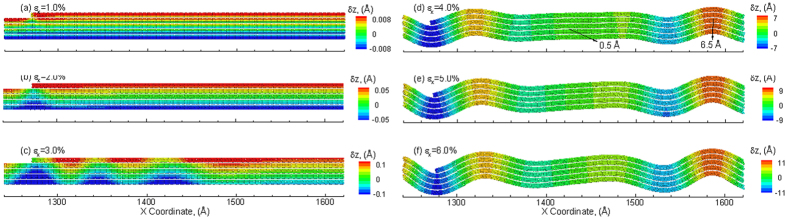
Snapshots of S5 showing propagation of ripples at the edge region at applied compressive strains of (**a**) *ε*_*x*_ = 1%, (**b**) *ε*_*x*_ = 2%, (**c**) *ε*_*x*_ = 3%, (**d**) *ε*_*x*_ = 4%, (**e**) *ε*_*x*_ = 5% and (**f**) *ε*_*x*_ = 6%. The atoms are colored by the displacement in the Z direction with the positive value (red color) corresponding to an upward displacement and a negative value (blue) indicating a downward displacement as compared to the unstrained configuration.

**Figure 8:**
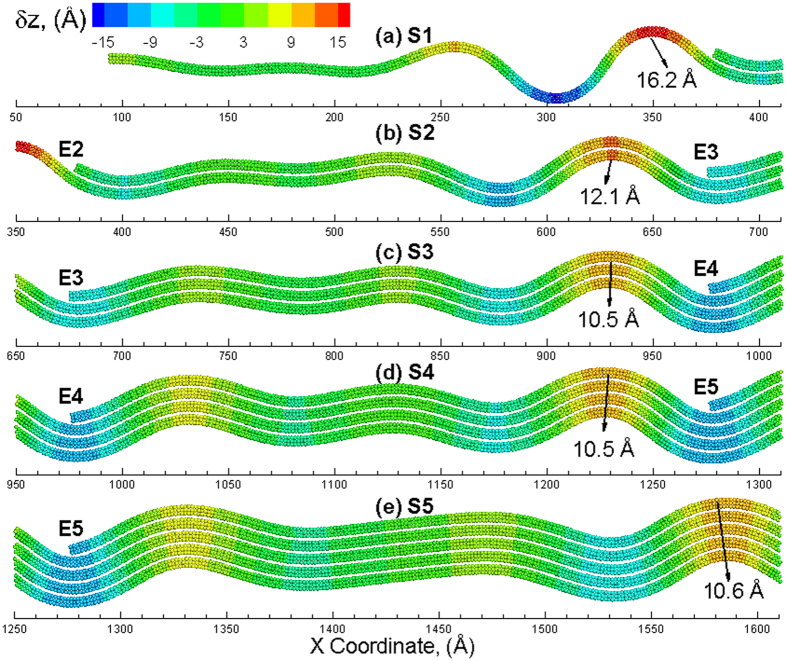
Snapshots of the sections (**a**) S1, (**b**) S2, (**c**) S3, (**d**) S4 and (**e**) S5 of 317 nm as-grown MoS_2_ structure at applied compressive strains of *ε*_*x*_ = 6%. The atoms are colored by the displacement in the Z direction with the positive value (red color) corresponding to an upward displacement and a negative value (blue) indicating a downward displacement as compared to the unstrained configuration.

The discussion so far has focused on the strain relaxation response under loading conditions of uniaxial tensile strain. The multilayered structure can also be subjected to compressive strains to investigate the strain response and ripple formation behavior. The variation of layer strain (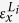) for each individual layer in multilayered structure as a function of applied uniaxial compressive strain (*ε*_*x*_) is shown in Fig. 6. The variation of 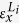 is initially linear with *ε*_*x*_. Similar to that observed for tensile strains, 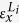 is observed to relax at larger applied strains through the nucleation and propagation of ripples. However, there exist a few noticeable differences in the case of compressive loading. The amount of layer strain relaxation in top layers during compression is considerably larger than that observed during tension. As will be discussed later, the larger relaxation of strain is attributed to the larger amplitudes of the ripples under compression as compared to that under tension. In addition, as the applied compressive strain exceeds *ε*_*x*_ = 3.75%, 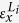 increases consistently with the applied strain. Such a re-emerged increase in the layer strain indicates that no additional strain is relaxed through additional nucleation of ripples. Thus, there is a limit for the relaxation of the layers that can be attributed to the formation of ripples. The vertical displacements in the section S5 of the pyramid structure at various values of applied strain of are shown in Fig. 7. The vertical displacements are fairly uniform in the section at an applied compressive strain of *ε*_*x*_ = 1% as shown in Fig. 7(a). Continued strain results in the nucleation of ripples at the edge E5 as shown in Fig. 7(b). Further applied strain nucleates more ripples that propagate towards the center of the structure as shown in Fig. 7(c). The most significant difference under compressive loading is observed at an applied compressive strain of *ε*_*x*_ = 4%, wherein the amplitude of the ripples exceeds 8 Å (~8.6 Å) at the edge of section S5. The amplitude, however, reduces to 6.5 Å at the center of the pyramid (right side of S5 in Fig. 7(d)) where the ripple initiated from the right edge merges with the left one. The maximum number of ripples is observed at an applied compressive strain of *ε*_*x*_ = 4%. Application of additional compressive strain does not result in nucleation of additional ripples. This can be seen from vertical displacements in Fig. 7(f) at an applied compressive strain of *ε*_*x*_ = 6% wherein the number of ripples propagating across section S5 is the same as those observed at an applied compressive strain of *ε*_*x*_ = 4%. The only difference at the two strains, however, is the amplitude of the ripples, which is implied by the different legends of the two snapshots. The vertical displacements for sections S1 to S5 at an applied compressive strain of *ε*_*x*_ = 6%, are shown in Fig. 8. Ripples are observed to nucleate at the edges for all the sections and propagate towards the center of the multilayered structure. The peak amplitude of the ripples in sections S1, S2, S3, and S5 are calculated to be 16.2 Å, 12.1 Å, 10.5 Å, 10.5 Å, 10.6 Å, respectively. These amplitudes of the ripples are in excellent agreement with the amplitudes of the ripples observed experimentally^34^. Also, the amplitude for sections with lower number of layers is observed to be larger, especially when the number of layers is less than three. The slightly larger amplitude in S5 (5 layers) as compared to that observed for section S4 (4 layers) is attributed to the interaction and merger of two ripples in S5.

While ripple formation has been reported using previous MD studies for the case of single layer MoS_2_ sheets^27^,^28^, the mechanism of ripple formation was attributed to buckling of the sheet under compression. The presence of edges in the multilayered structure considered here provides the necessary displacements to relax strain through the formation of ripples. The presence of terraced edges in these CVD-grown structures is observed to be the cause for the nucleation of ripples at the edges that grow towards the center of the structure under applied in-plane strains. To verify this, a five-layered MoS_2_ sheet with the same dimensions as section S5 of the multilayered structure is created with equal dimensions of all layers (i.e. no edges) and is subjected to uniaxial compression as discussed before. The multilayered MoS_2_ structure comprises of 317 nm in X direction and 10.98 nm in y direction, which is of same size as the bottom layer in 317 nm as-grown MoS_2_ multilayer. The top four layers are of the same dimensions as bottom layer in this multilayered MoS_2_ structure. The MD simulations of uniaxial tensile strain indicate that no strain relaxation is observed in the periodic sheet and the layer strain 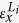
for each layer is observed to the be the same as the applied strain for all the layers up to an applied strain of *ε*_*x*_ = 4%. Thus, no ripples are generated without the presence of the edges, verifying the primary role of edges in ripple formation and strain relaxation of as-grown multilayered structures.

Another factor to consider in the role of edges on the formation of ripples is the size of the terrace structures between the edges. As a result, two multilayered MoS_2_ systems are created with the same ratio of the lengths of the layers, but with the dimensions of ~127 nm for the bottom layer (defined as the small system) and ~634 nm for the bottom layer (defined as the large system). Simultaneous relaxation is noticeably observed in 127 nm system and slightly in 634 nm system. For both cases, 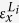 in the bottom layer is identical to 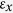, and top four layers relax to various extent. The relaxation strains of the top layer (fifth layer) are calculated to be 1.85%, 2.75% and 3.75% for the systems with dimensions of 127 nm, 317 nm and 634 nm, indicating the strains required to nucleate ripples increases for the larger dimensions of the terraces.

A terrace edge-to-bulk ratio (EBR) ratio can be defined for the layers in 2D MoS_2_ structure to describe the significance of edges in strain relaxation. For the multilayered structure considered here that is periodic in the Y direction, it is evident that a larger length in X direction will result in a smaller EBR, thus decreasing edge effect. It is revealed from Fig. 2 and Figure S6 that the values of the 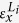 decrease from bottom to the top layer, which is attributed to the diminishing EBR. The atoms in the top layers tend to relax to a larger extent due to more severe edge effect that results in vertical displacements and formation of ripples. Similarly, the layers in a larger sized pyramid have a smaller EBR, thus a smaller amount of strain reduction.

## Conclusions

Molecular dynamics simulations are carried out to investigate the strain response of suspended CVD-grown MoS_2_ structures at the atomic scales. To investigate the strain response, a model multilayered system with terraces and edges is created to mimic the CVD-grown system. The MD simulations using a hybrid REBO/LJ potential suggest that the strain response of a suspended multilayered system is layer dependent and the relaxation of individual layers is attributed to nucleation, propagation and interaction of ripples. The ripples are observed to nucleate at the locations of the edges in the regions below the edge and propagate inward to the center of the multilayered structure. The ripples formed have vertical displacements of ~0.1 Å for loading under uniaxial tensile strain and ~10 Å for loading under uniaxial compressive strain. The strains required for the formation of ripples is observed to be dependent on the dimensions of the layers. It should be pointed out that current study is performed without a substrate so that the model is simplified enough to reveal the underlying origins of the ripples in suspended terraced MoS_2_ structures. As a result, the effects of substrate are not considered. For the case of MoS_2_ flakes or terraced few-layered MoS_2_ flakes on a substrate, the interaction between substrates and MoS_2_ layers will affect the rippling behavior significantly as the presence of a substrate limits the vertical displacements and the strain relaxation behavior will be determined by in-plane displacements. The formation of ripples in CVD-grown few-layered structures under strain need to be accounted for when analyzing strain effects on electronic properties of these as-grown structures materials using experimental methods or in continuum models. The strain response of suspended CVD-grown structures through ripple formation also shows promise for the use of strain engineering to generate inhomogeneous electronic properties for the design novel devices based on CVD-growth MoS_2_ structures.

## Methods

### Density Functional Theory

DFT calculations are carried out using projector augmented wave pseudo-potentials^39^ as implemented in the VASP code^40^. The cutoff energy is 500 eV for the plane-wave expansions and the Monkhorst–Pack *k*-point mesh is 17 × 17 × 1. The exchange-correlation functional is treated within the Perdew–Burke–Ernzerhof (PBE) generalized gradient approximations (GGA)^41^. The conventional DFT energy is supplemented with a pairwise interatomic vdW potential which is determined by Tkatchenko and Scheffler (TS-vdW) from non-empirical mean-field electronic structure calculations^42^. The atomic positions of a unit cell (3 atoms) are optimized until all components of the forces on each atom are reduced to values below 0.01 eV/Å.

### Molecular Dynamics

Large-scale MD simulations of model as-grown systems are carried out using LAMMPS^43^ with the interatomic interactions defined using the reactive empirical bond-order potential^36^ combined with a Lennard Jones potential. The time step for all simulations is defined to be 1 fs. All systems created are first equilibrated at 0 K for 10 ps at constant temperature and zero pressure (NPT ensemble using the Nose-Hoover algorithm), then the equilibrated structure is used as a strain free configuration for the following deformation simulations.

### Interatomic Potentials

The reactive empirical bond-order (REBO) potential is chosen to describe in-plane covalent interaction of Mo-S system, because it is able to provide an accurate description of the structural energetics for bulk MoS_2_ (the in-plane lattice parameter *a*, the vertical S-S distance *d*, and the intra-layer lattice parameter *c*) as well as the bond breaking and formation processes at extreme tensile and compressive stresses^44^,^45^. The out-of-plane van der Waals (vdW) interaction between adjacent layers is described by pairwise Lennard Jones (LJ) potential. The energy for an atom in the REBO formulation is calculated as:


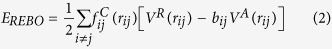


where 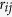 is the distance between atoms *i* and *j*, 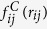 is a cutoff function and smoothly switches REBO to LJ. *V*^*R*^(*r*_*ij*_) and *V*^*A*^(*r*_*ij*_) are repulsive and attractive portion, respectively.


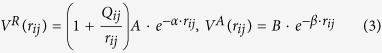


The intralayer vdW interaction is described by the LJ potential:


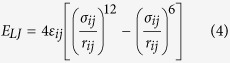


To demonstrate the capability of the combined potential to model the strain response of the CVD-grown MoS_2_ structures, MD simulations are first carried out to reproduce the strain response of bilayer MoS_2_ structure with free edge as observed using DFT simulations^33^. The bilayer edge structure is shown in Figure S1. The system in the 2*H* stacking sequence is rectangular in the basal (XY) plane with the thickness of the two layers to be equal (Y direction), but relatively smaller along the X direction. Three dimensional periodic boundary conditions are applied in the calculations so that the bottom MoS_2_ layer is actually infinitely large along X and Y directions, whereas the top layer has a finite width in the X direction and infinitely long along the Y direction. A vacuum space of 20 Å thick is used along the vertical direction of the system to prevent unphysical interactions between adjacent images along this direction. The local strain maps^33^ under an applied uniaxial strain of *ε*_*x*_ = 4% as computed using DFT and MD simulations are shown in Figure S2. It can be seen that the MD simulations using the combined REBO-LJ potential are able to reproduce the strain variations in the two layers as predicted using DFT simulations^33^. The strain in the top layer is observed to be significantly lower as compared to the bottom layer that corresponds to the value of the applied strain (around 4%). Moreover, both methods show that the residual strain in the edge region of the top layer is smaller than the center region, while the opposite trend is observed for the bottom layer.

## Additional Information

**How to cite this article**: Wang, J. *et al*. Origins of Ripples in CVD-Grown Few-layered MoS_2_ Structures under Applied Strain at Atomic Scales. *Sci. Rep.* **7**, 40862; doi: 10.1038/srep40862 (2017).

**Publisher's note:** Springer Nature remains neutral with regard to jurisdictional claims in published maps and institutional affiliations.

## Supplementary Material

Supplementary Figures
