# Supplementary material for: Origins of Ripples in CVD-Grown Few-layered MoS2 Structures under Applied Strain at Atomic Scales
Source: Sci Rep. 2017 Jan 19;7:40862. doi: 10.1038/srep40862 (PMC5244384; doi:10.1038/srep40862)
Supplement: Supplementary Figures [file srep40862-s1.pdf]

SUPPLEMENTAL FIGURES AND FIGURE CAPTIONS

Title: Origins of Ripples in CVD-Grown Few-layered MoS_2 Structures under Applied Strain at Atomic Scales

Authors: Jin Wang[1], Raju R. Namburu[2], Madan Dubey[3], and Avinash M. Dongare[1],*

1 Department of Materials Science and Engineering and Institute of Materials Science, University of Connecticut, Storrs, Connecticut 06269, USA

2 Computational and Information Sciences Directorate, U.S. Army Research Laboratory, Aberdeen Proving Ground, Maryland 21005, USA

3 Sensors and Electron Devices Directorate, U.S. Army Research Laboratory, Adelphi, Maryland 20783, USA

*** Corresponding Author:**

Avinash M. Dongare

Materials Science and Engineering, University of Connecticut, Storrs, CT-06084

Email: dongare@uconn.edu

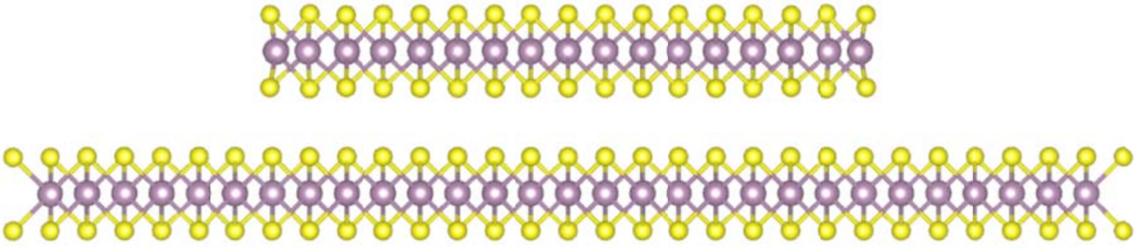

Figure S1: Schematic show of the bilayer supercell used in this study.

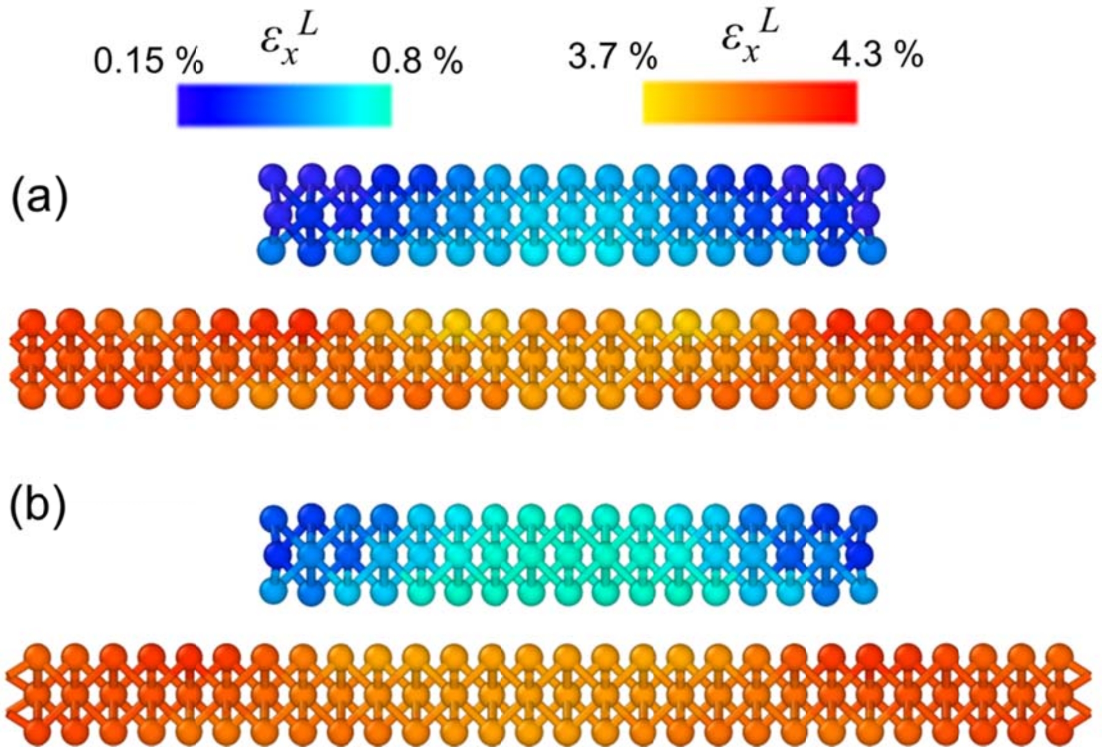

Figure S2: Comparison of (a) DFT and (b) MD in local strain (ε_x^L) map of a bilayer MoS_2 system under an applied uniaxial strain ε_x=4%.

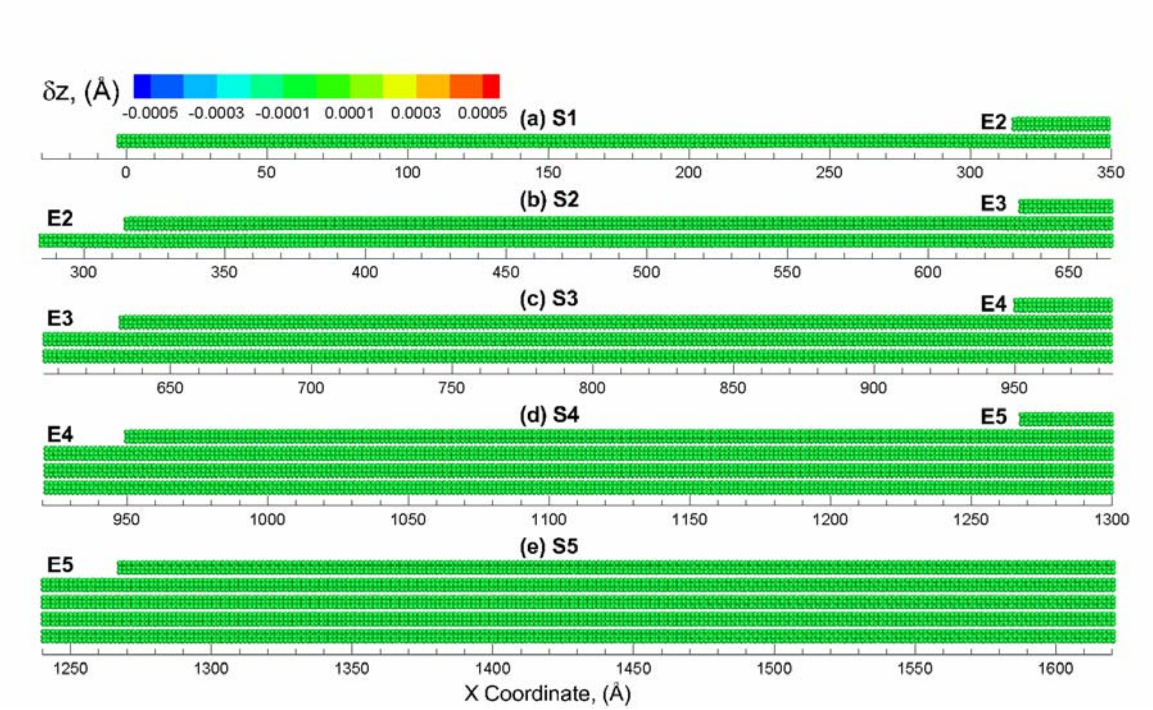

Figure S3: The vertical displacement map in the unstrained configuration at different sections: (a) S1, (b) S2, (c) S3, (d) S4 and (e) S5. The atoms are colored by the displacement in the Z direction with the positive value (red color) corresponding to an upward displacement and a negative value (blue) indicating a downward displacement as compared to the unstrained configuration.

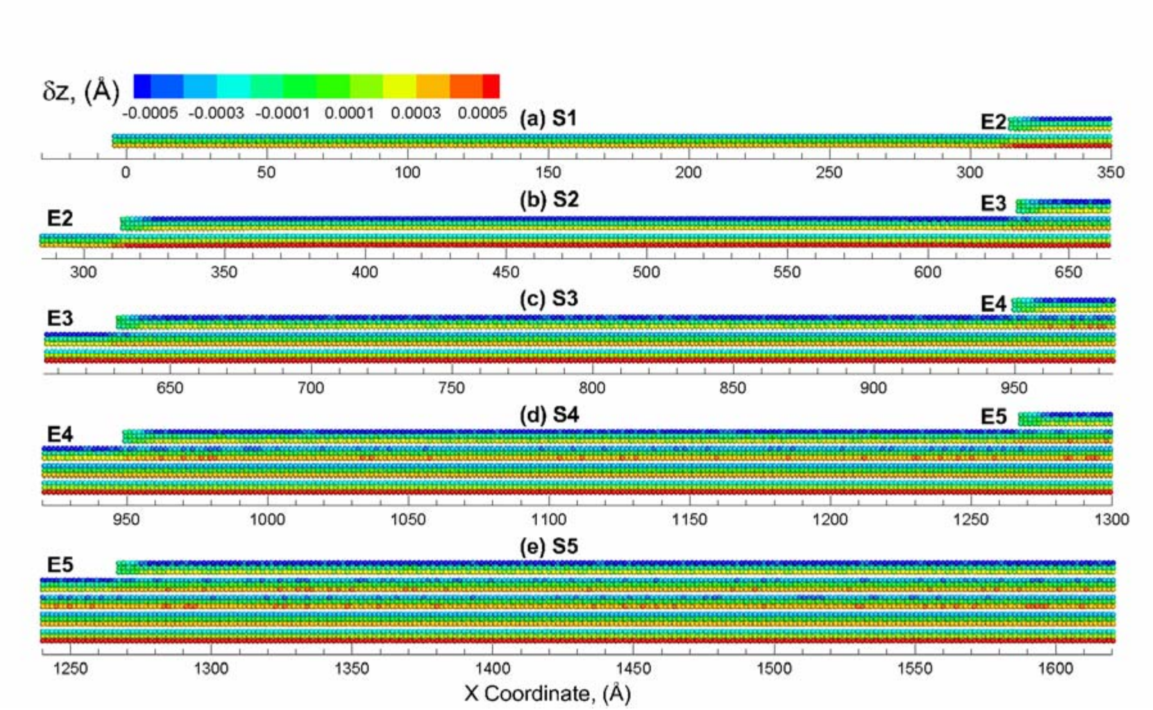

Figure S4: The variation of the vertical displacement in the various sections (a) S1, (b) S2, (c) S3, (d) S4 and (e) S5 of the multilayered structure at an applied strain of $\varepsilon_x = 0.1$ %. The atoms are colored by the displacement in the Z direction with the positive value (red color) corresponding to an upward displacement and a negative value (blue) indicating a downward displacement as compared to the unstrained configuration.

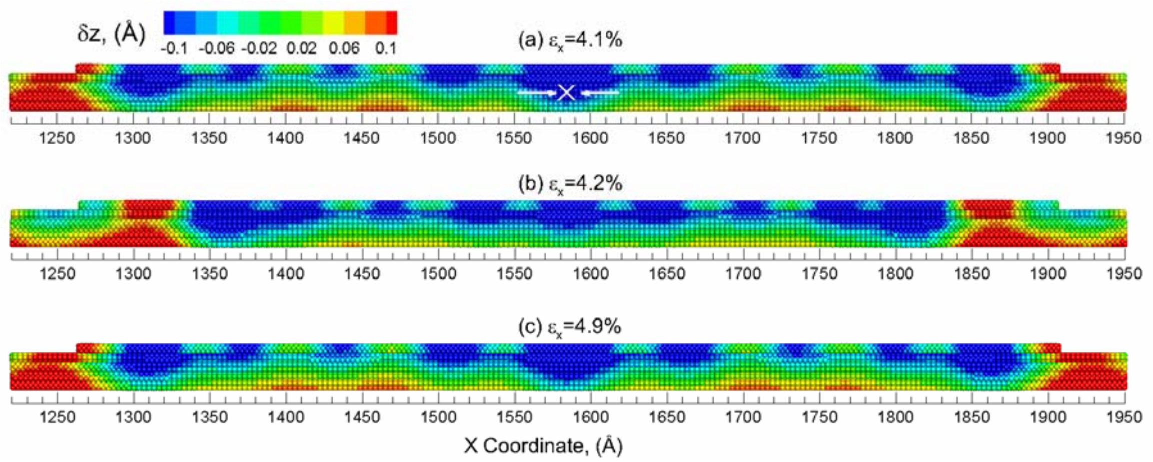

Figure S5: Snapshots of center of pyramid system at various strains (a) ε_x = 4.1%, (b) ε_x = 4.2% and (b) ε_x = 4.9% showing propagation of ripples at the edge region. The atoms are colored by the displacement in the Z direction with the positive value (red color) corresponding to an upward displacement and a negative value (blue) indicating a downward displacement as compared to the unstrained configuration.

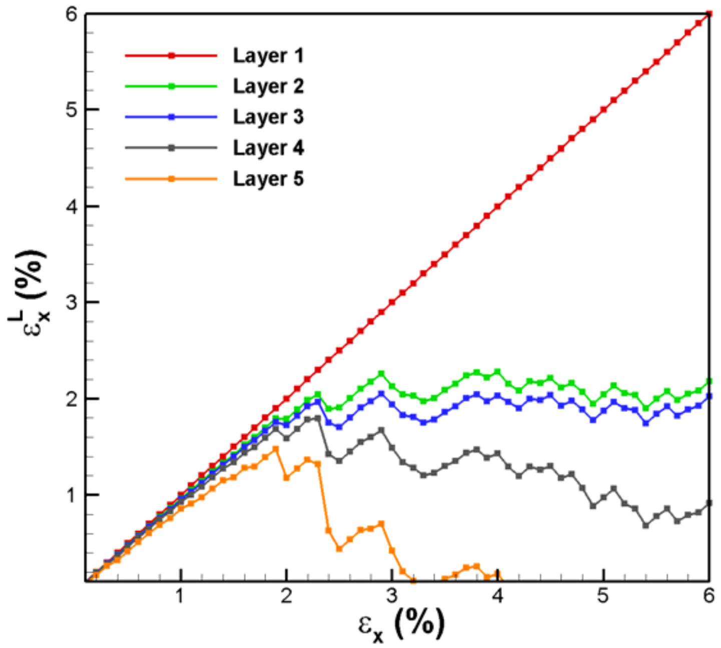

(a)

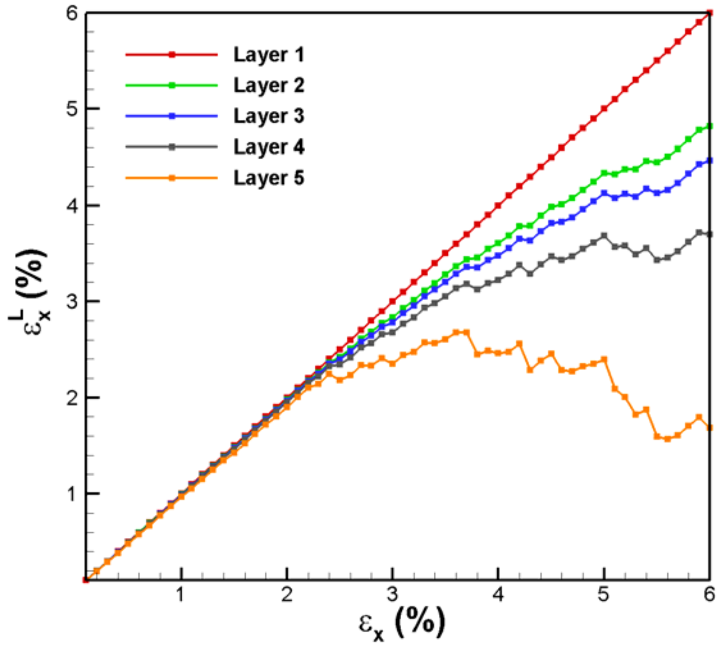

(b)

Figure S6: Local layer strain ε_x^L as a function of applied strain ε_x for (a) 127 nm, (b) 634 nm as-grown multilayered MoS_2 structure under uniaxial tensile strain.
